# Localized Path Planning for Mobile Robots Based on a Subarea-Artificial Potential Field Model

**DOI:** 10.3390/s24113604

**Published:** 2024-06-03

**Authors:** Qiang Lv, Guoqiang Hao, Zhen Huang, Bin Li, Dandan Fu, Huanlong Zhao, Wei Chen, Sheng Chen

**Affiliations:** 1 School of Electrical and Electronic Engineering, Wuhan Polytechnic University, Wuhan 430048, Chinahuanlzhao@outlook.com (H.Z.);; 2 College of Mechanical Engineering, Wuhan Polytechnic University, Wuhan 430023, China; libin_027@126.com (B.L.);

**Keywords:** artificial potential field, predicted potential field, subarea potential field, local path planning, autonomous mobile robot, path planning, obstacle avoidance

## Abstract

The artificial potential field method has efficient obstacle avoidance ability, but this traditional method suffers from local minima, unreasonable paths, and sudden changes in heading angles during obstacle avoidance, leading to rough paths and increased energy consumption. To enable autonomous mobile robots (AMR) to escape from local minimum traps and move along reasonable, smooth paths while reducing travel time and energy consumption, in this paper, an artificial potential field method based on subareas is proposed. First, the optimal virtual subgoal was obtained around the obstacles based on the relationship between the AMR, obstacles, and goal points in the local environment. This was done according to the virtual subgoal benefit function to solve the local minima problem and select a reasonable path. Secondly, when AMR encountered an obstacle, the subarea-potential field model was utilized to solve problems such as path zigzagging and increased energy consumption due to excessive changes in the turning angle; this helped to smooth its planning path. Through simulations and actual testing, the algorithm in this paper demonstrated smoother heading angle changes, reduced energy consumption, and a 10.95% average reduction in movement time when facing a complex environment. This proves the feasibility of the algorithm.

## 1. Introduction

In recent years, with the rapid development of intelligent control and artificial intelligence, AMRs have been applied in fields such as mineral exploration, military reconnaissance, cargo handling, and industrial production [1]. During the execution of tasks, AMRs encounter various obstacles, and the question of how to plan a reasonable path to enable them to efficiently avoid obstacles and reach their destination safely has become one of the hot topics in AMR path-planning technology [2].

AMR path planning can be divided into two categories [3]: The first category is global path planning, where the entire driving path is determined based on the complete prior information of the environmental map [4]. Examples of algorithms used in global path planning include the A* algorithm [5,6], Dijkstra algorithm [7], genetic algorithm [8], and Rapidly-exploring Random Tree [9], among others. However, the effectiveness of the planned path in global path planning is limited by the accuracy and non-real-time updating of the pre-loaded environment map [10]. The second category is local path planning, which is based on partial knowledge of the environment along with real-time information obtained from sensors mounted on the AMR to dynamically plan the driving path [11]. Examples of algorithms used in local path planning include the dynamic window approach [12], velocity obstacle method [13], artificial potential field method [14], and reinforcement learning [15], among others.

In local path planning, the artificial potential field method is widely applied [16]. The artificial potential field method treats the task environment of the AMR as a potential field. In this method, the goal point generates an attractive potential field to guide the AMR toward the goal, while obstacles in the environment generate repulsive potential fields to keep the AMR away from obstacles [17]. However, traditional artificial potential field methods face challenges such as local minima and excessive deviation in the heading angle, leading to unrealistic paths [18]. Therefore, many scholars have made improvements to address the issues of the traditional artificial potential field method [19]. When faced with local minima situations, Szczepanski et al. [20] predicted the upcoming local minima points in advance to guide the AMR around them and reach the goal. However, their approach suffered from abrupt changes in turning angles and non-smooth path planning. Wu et al. [21] proposed a deterministic annealing-based artificial potential field method to prevent the AMR from getting trapped in local minima traps. However, their approach exhibited discontinuous changes in turning angles during path planning. Guo et al. [22] created guide points around obstacles to provide additional attraction for the AMR to escape local minimum situations. Hossain et al. [23] used a dynamic window and improved follow-the-gap method to calculate reasonable deviation angles for the goal points, enabling the AMR to reach the goal in the presence of dynamic obstacles. However, their approach suffered from excessive turning angles in path planning. Hu et al. [24] optimized the planned path using Said–Ball curves, resulting in smooth paths in scenarios with multiple obstacles. Yang et al. [25] introduced a smoothing switch function and optimized the repulsive potential field parameters to achieve smoother trajectories for the mobile robot. Wang et al. [26] combined genetic algorithms with artificial potential fields and introduced B-spline smoothing to modify the globally planned path, resulting in smoother turning angles in the trajectory.

To address issues such as local minima and unreasonable paths in path planning, this paper proposes a local path-planning strategy for mobile robots based on the Subarea-Artificial Potential Field Model (S-APFM). Firstly, a virtual subgoal utility function is designed to select the optimal virtual subgoal around obstacles, effectively solving the problems caused by local minima and the irrational placement of virtual subgoals leading to path elongation. Secondly, the Subarea-Potential Field Model (S-PFM) is introduced to smooth the changes in heading angles during obstacle avoidance, resulting in smoother planned paths.

The organizational structure of this article is as follows: Section 2 introduces the local path planning strategy for mobile robots based on the Subarea-Artificial Potential Field Model. Section 3 provides a detailed description of the algorithm simulation and results analysis. Section 4 covers the experimental results and analysis. Section 5 discusses the local path planning strategy. Section 6 summarizes this article and presents outlooks for the future.

## 2. Local Path Planning Strategy for Mobile Robots Based on the Subarea-Artificial Potential Field Model

**Figure 1:**
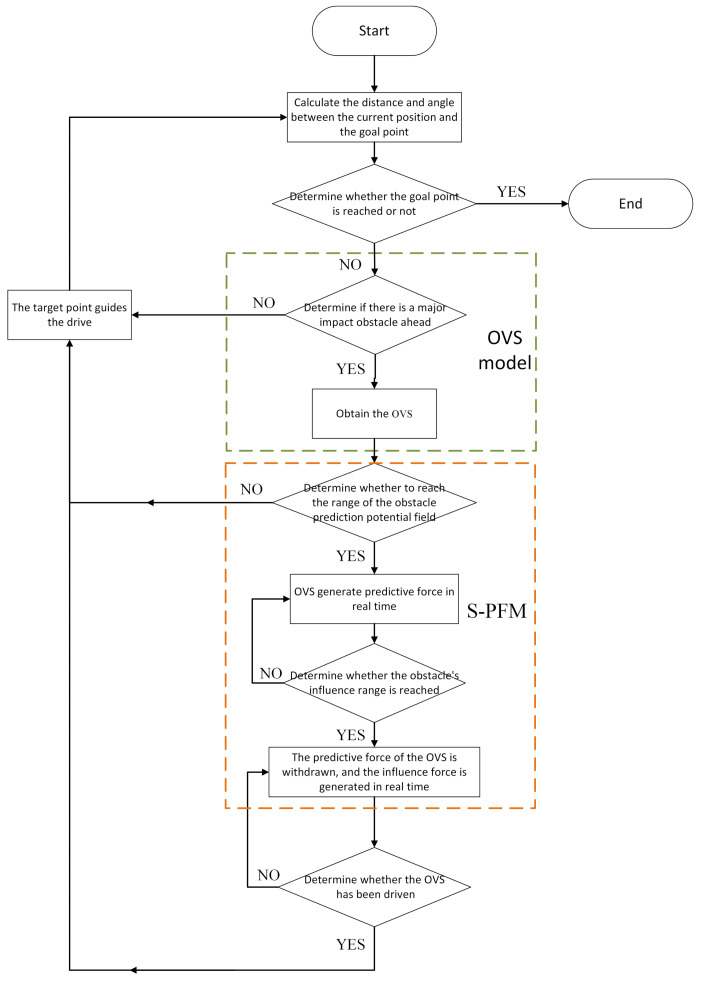
S-APFM algorithm flow chart.

The S-APFM algorithm flow is shown in Figure 1. First, the main influencing obstacles in front of the AMR were identified based on calculations. Then, the optimal virtual subgoals (OVS) were obtained using the optimal virtual subgoal model. Finally, when the AMR reached the range of the subregion potential field of the main influencing obstacle, the S-PFM was utilized to smoothly navigate around the obstacle and reach the goal point. The S-APFM algorithm involves two key technical points: constructing the optimal virtual subgoal model and building the S-PFM.

### 2.1. Construction of Optimal Virtual Subgoal Model

During the motion of the AMR, obstacles are avoided and local minima traps are escaped. Firstly, calculations affect the path into obstacles (APO). Secondly, the pending virtual subgoals (PVS) are set around the APO [27]. Finally, the optimal virtual subgoal (OVS) is calculated using the virtual subgoal utility function J, and the AMR is guided to move towards the OVS to avoid obstacles.

#### 2.1.1. Set Pending Virtual Subgoals Based on Collision Detection

**Figure 2:**
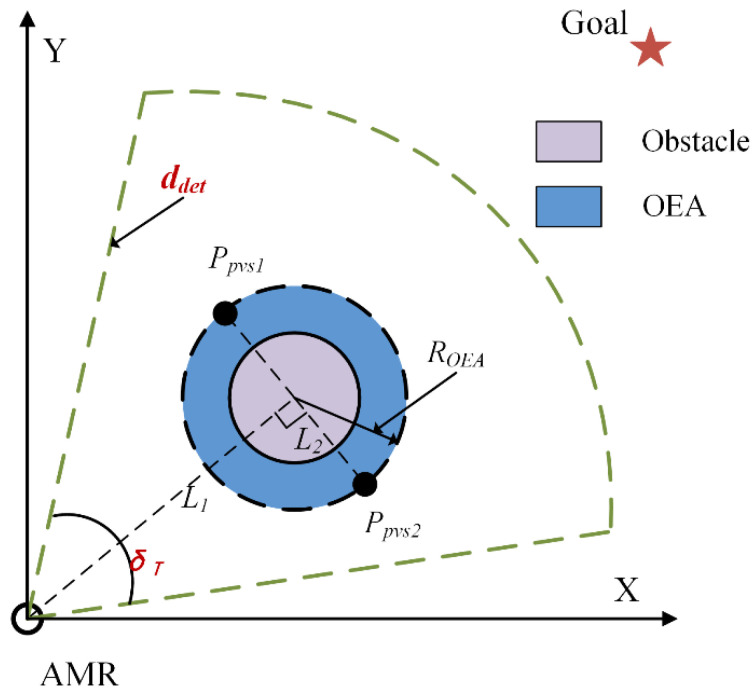
PVS setup diagram.

The current investigation introduced collision detection [23,28,29,30,31] and set the detection distance *d_det_* and detection angle *δ_T_* according to the sensor characteristics of the AMR [20,28], calculating the APO on the AMR’s driving path. Meanwhile, to reduce the risk of collision, an Obstacle Expansion Area (OEA) with a radius of *R_OEA_* was set in the area around the APO [23,32] (Obstacle Expansion Area, OEA), as shown in Figure 2. The calculation of *R_OEA_* is shown in Equation (1) [22,28,29]. To simplify the schematic diagram, the AMR is treated as a particle in this article.

(1) $$R_{OEA}=R_{obs}+d_{AMR},$$

where *R_obs_* represents the radius of the obstacle and *d_AMR_* represents the diameter of the AMR.

Secondly, the PVS is selected around the APO for the subsequent screening of the OVS [27], as shown in Figure 2. *L*_1_ is the line connecting the AMR and the center of the obstacle; *L*_2_ is perpendicular to *L*_1,_ with the intersection at the center of the obstacle, intersecting the outer boundary of OEA at points *P_pvs_*_1_ and *P_pvs_*_2_. These two points are the PVS [28].

#### 2.1.2. Using the Benefit Function to Determine the Optimal Virtual Subgoal

A reasonable virtual subgoal can reduce the path length and energy consumption of AMR during operation. Therefore, it is crucial to select OVS from the PVS for the subsequent travel of the AMR. To screen the OVS, this study designed a virtual subgoal benefit function J based on the distance and angle relationship between the PVS and obstacles. The utility benefit function J for the virtual subgoal is shown in Equation (2):

(2) $$J=\begin{array}{cc} e^{V_{dist}/V_{angle}} & 0<d_{Aob}\leq d_{\det} \end{array},$$

where *d_Aob_* represents the distance between the AMR and the obstacle. *V_dist_* represents the distance parameter and *V_angle_* represents the angle parameter.

**Figure 3:**
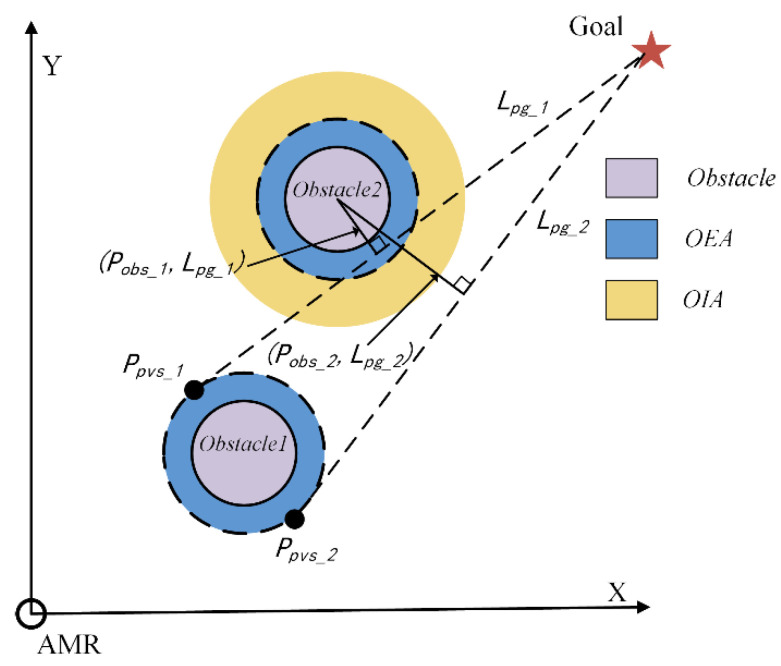
PVS and obstacle distance relationship diagram.

(1) Distance parameter *V_dist_*:

Considers the presence of obstacles between the APO and the goal point, as shown in Figure 3. An Obstacle Influence Area (OIA) is introduced outside the OEA due to the presence of obstacles [33], with a radius of *R_OIA_*. The numerical range of *R_OIA_* is 1.85 *R_obs_* to 2.85 *R_obs_*. The value of *R_OIA_* affects the size of the obstacle avoidance range of the AMR. *L_pg_* represents the line connecting the PVS and the goal point, and (*P_obs_*, *L_pg_*) represents the distance from the obstacle to *L_pg_*.

The formula for calculating the distance parameter *V_dist_* is shown in Equations (3) and (4):

(3) $$V_{dist}=1/e^{V_{\sigma }},$$

(4) $$V_{\sigma }=\sum_{k=1}^{m}\sum_{i=1}^{n}\frac{\left((P_{obs\_k},L_{pg\_i})-R_{OIA}\right)}{R_{OEA}}\;\begin{array}{c} \left(n=2;k=1,2,\ldots m\right) \end{array},$$

where *n* represents the number of pending virtual subgoals and m represents the number of obstacles between the APO and the goal point. When the value of ((*P_obs_, L_pg_*) − *R_OIA_*) is smaller, this means that *L_pg_* is closer to the OIA, the larger the *V_dist_*. Therefore, the AMR needs to perform obstacle avoidance actions to move away from the obstacles in the OIA region. Hence, a larger V*_dist_* has a greater impact on the subsequent path of the AMR.

(2) Angle parameter *V_angle_*

**Figure 4:**
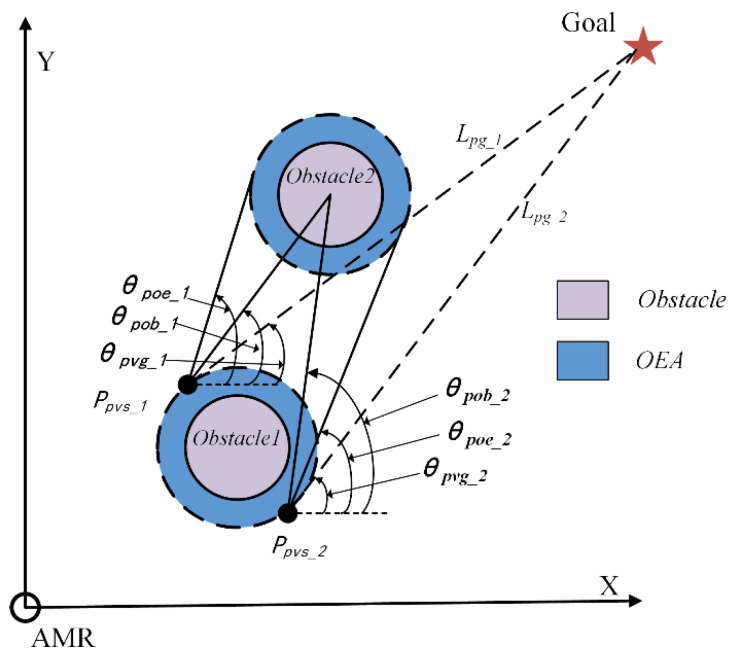
PVS and obstacle Angle diagram.

The angle parameter *V_angle_* takes into account the angle relationship between the PVS, obstacles, and the goal point, as shown in Figure 4. *θ_poe_* represents the tangent angle from the PVS to OEA, *θ_pob_* represents the angle from the PVS to the center of the obstacle, and *θ_pvg_* represents the angle from the PVS to the line connecting to the goal point.

The calculation of *V_angle_* is shown in Equation (5):

(5) $$V_{angle}=\left\{\begin{array}{l} \sum_{k=1}^{m}\sum_{i=1}^{n}\lambda (\frac{\theta_{pob\_k}-\theta_{pvg\_i}}{\left|\theta_{poe\_i}-\theta_{pob\_k}\right|})\begin{array}{c} \begin{array}{cc} \theta_{pob\_k}>\theta_{pvg\_i} & \left(n=2;k=1,2\cdots m\right) \end{array} \end{array}\\ \sum_{k=1}^{m}\sum_{i=1}^{n}\lambda (\frac{\theta_{pvg\_i}-\theta_{pob\_k}}{\left|\theta_{poe\_i}-\theta_{pob\_k}\right|})\begin{array}{c} \begin{array}{cc} \theta_{pob\_k}\leq \theta_{pvg\_i} & \left(n=2;k=1,2\cdots m\right) \end{array} \end{array} \end{array}\right.,$$

where *λ* is added as a constant to avoid extremely small values for *V_angle_*. When $\frac{\theta_{pvg\_i}-\theta_{pob\_k}}{\left|\theta_{poe\_i}-\theta_{pob\_k}\right|}$ is smaller, indicating a smaller *V_angle_,* this means that *L_pg_* is closer to the line connecting the center of PVS and the obstacle. This implies that the AMR needs to avoid the obstacle at a larger angle in the subsequent path. Therefore, a smaller *V_angle_* has a greater impact on the subsequent path of the AMR.

**Figure 5:**
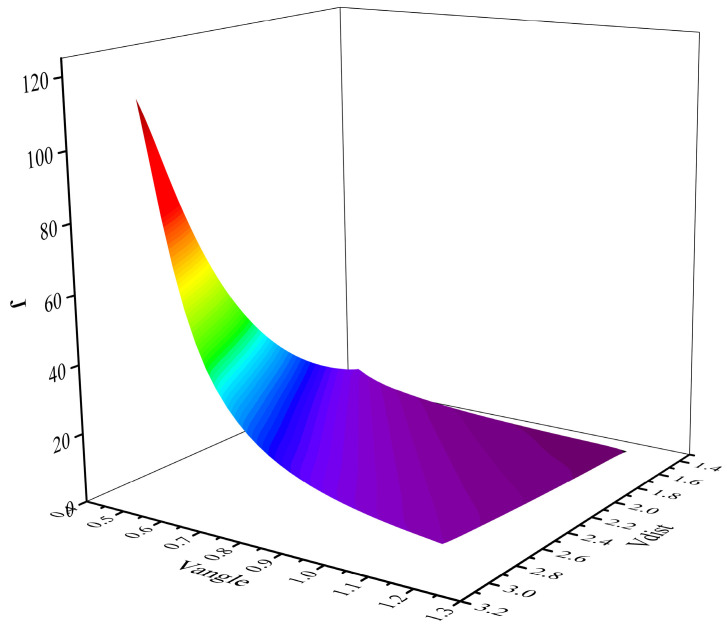
Relationship diagram between *J* and *V_dist_* and *V_angle_*.

The virtual subgoal benefit function *J* varies with *V_dist_* and *V_angle_*, as shown in Figure 5. As *V_dist_* increases, *V_angle_* decreases and J increases.

### 2.2. Subregion-Potential Field Model

After the selection of the OVS was completed, the AMR still faced the problem of excessive changes in the heading angle during obstacle avoidance [34], which results in an unsmooth planned path. The smoothness of the path has a significant impact on the process of AMR’s obstacle avoidance [35].

**Figure 6:**
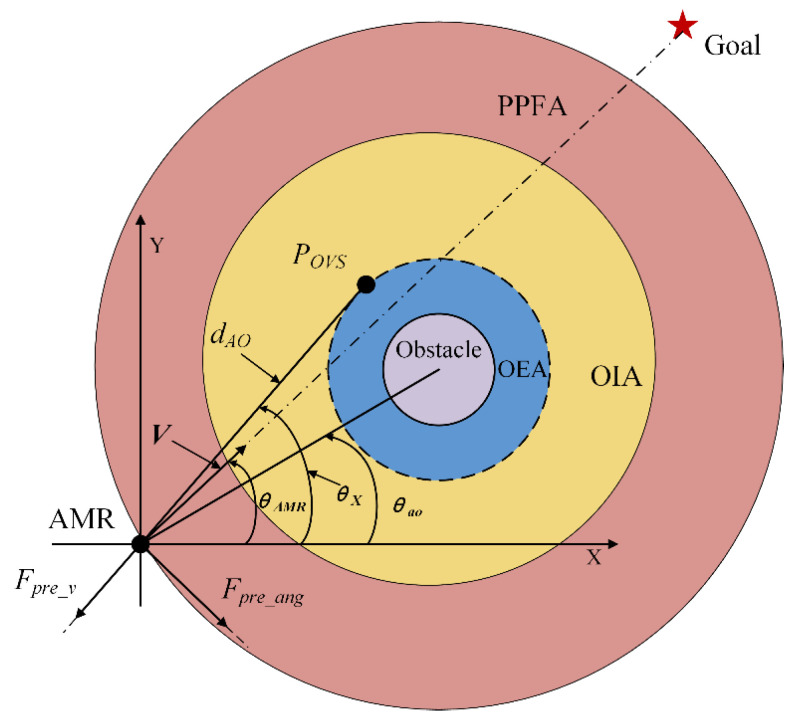
Predicted potential field diagram.

To reduce the angle changes and obtain a smoother path for the AMR during obstacle avoidance, this study introduced the Predicted Potential Field [36], virtual subgoal influence force, and angle constraints to form a Subarea-Potential Field Model (S-PFM) [30,36,37]. At the same time, a series of circular regions were set around the obstacles, as shown in Figure 6. Starting from the innermost region and moving outward, these circular regions were the Obstacle Expansion Area (OEA), the Obstacle Influence Area (OIA), and the Predicted Potential Field Area (PPFA) [28,33,38]. The Predicted Potential Field exerted its influence in the PPFA circular region, enabling the anticipation and control of the AMR’s avoidance angle. The influencing force of the virtual subgoal was applied within the OIA circular region.

#### 2.2.1. Predictive Force within the Predicted Potential Field Area (PPFA)

As the AMR is not only influenced by its previous velocity while traveling but is also affected by the direction of the potential field force, this paper introduced predictive force to proactively control the avoidance angle of the AMR [36]. At the same time, the predicted potential field force within the PPFA circular region was decomposed into the velocity predictive force ${\overset{⃑}{F}}_{pre\_v}$ and the angle predictive force ${\overset{⃑}{F}}_{pre\_ang}$, with the two forces being perpendicular to each other [36,39]. This is shown in Figure 6, where V represents the current velocity of the AMR, and where *P_OVS_* is the optimal virtual subgoal.

The magnitude of the velocity predictive force ${\overset{⃑}{F}}_{pre\_v}$ was calculated as shown in Equation (6), and the magnitude of ${\overset{⃑}{F}}_{pre\_v}$ was determined by the angle between the AMR and PVS:

(6) $$F_{pre\_v}=k_{pre\_ve}\exp(\frac{-\theta_{X}+\theta_{AMR}}{\left|\theta_{ao}-\theta_{X}\right|}-1),$$

where *k_pre_ve_* represents the velocity prediction force gain coefficient, *θ_AMR_* represents the current velocity direction of the AMR, *θ_X_* represents the angle between the AMR and the line connecting it to the OVS, and *θ_ao_* represents the angle between the AMR and the line connecting it to the center of the obstacle. The direction of the velocity prediction force ${\overset{⃑}{F}}_{pre\_v}$ is collinear with the line connecting the AMR and OVS.

The magnitude of the angle predictive force ${\overset{⃑}{F}}_{pre\_ang}$ is calculated as shown in Equation (7). The magnitude of ${\overset{⃑}{F}}_{pre\_ang}$ is defined by the distance between the AMR and the PVS. When the AMR approaches the PVS, the current motion angle should be adjusted to control the direction of obstacle avoidance.

(7) $$F_{pre\_ang}=k_{pre\_ang}\exp(\frac{d_{\mathrm{AO}}}{R_{OEA}}-1),$$

where *k_pre_ang_* represents the gain coefficient of the angle predictive force and *d_AO_* represents the distance between the AMR and the *P_OVS_*. The direction of the angle predictive force ${\overset{⃑}{F}}_{pre\_v}$ is perpendicular to the line connecting the AMR and the PVS.

When the AMR is within the PPFA circular region, in addition to being affected by the predictive potential field force, it is also subject to the attraction force ${\overset{⃑}{F}}_{att}(goal)$ of the goal point [17]. The magnitude of ${\overset{⃑}{F}}_{att}(goal)$ is calculated as shown in Equation (8):

(8) $$F_{att}(goal)=k_{att}d(P_{AMR},P_{goal}),$$

where *k_att_* represents the attraction gain coefficient and *d*(*P_AMR_*, *P_goal_*) represents the distance between the current position of the AMR and the goal point. The direction of the attraction force ${\overset{⃑}{F}}_{att}(goal)$ is from the AMR towards the goal point.

Therefore, when the AMR is within the PPFA, the total force ${\overset{⃑}{F}}_{pre\_all}$ acting on it is given by Equation (9):

(9) $${\vec{F}}_{pre\_all}={\vec{F}}_{att}(goal)+{\vec{F}}_{pre\_ang}+{\vec{F}}_{pre\_v},$$

#### 2.2.2. Influence Force of Virtual Subgoals in the Obstacle Influence Area (OIA)

**Figure 7:**
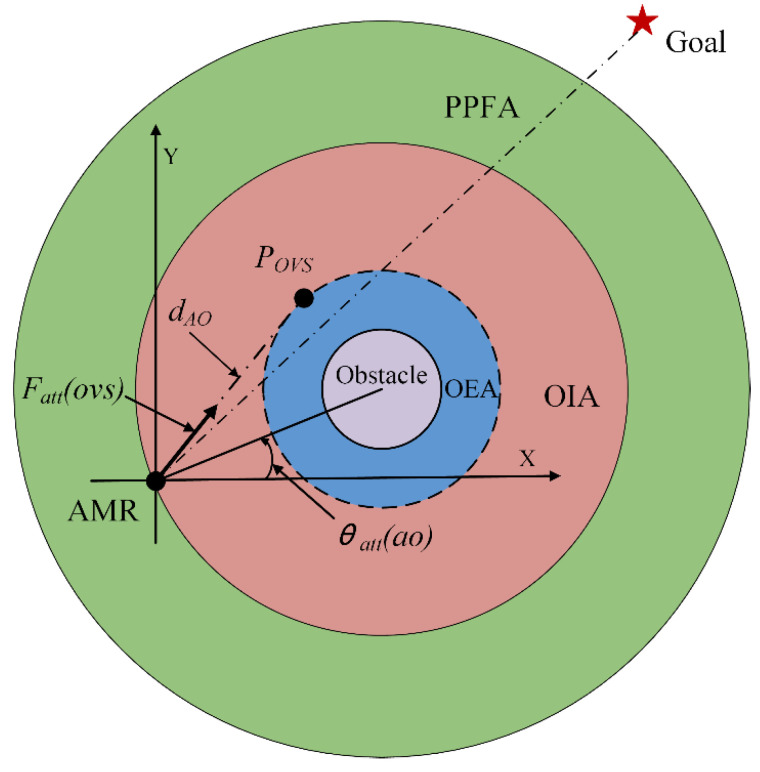
OVS influence force diagram.

When the AMR travels through the PPFA circular region and enters the OIA circular region, the force ${\overset{⃑}{F}}_{pre\_all}$ is canceled out, and it is only influenced by the influence force ${\overset{⃑}{F}}_{att}(ovs)$ generated by the obstacle avoidance potential field of the OVS [17]. This guides the AMR to avoid obstacles within the OIA, as illustrated in Figure 7.

The magnitude of the influence force ${\overset{⃑}{F}}_{att}(ovs)$ acting on the AMR within the OIA is given by Equation (10), as shown [17]:

(10) $$F_{att}(ovs)=k_{vs\_att}d_{AO},$$

where *k_vs_att_* represents the improved attraction potential coefficient.

The magnitudes of the two component forces ${\overset{⃑}{F}}_{att\_x}(ovs)$ and ${\overset{⃑}{F}}_{att\_y}(ovs)$ of the force ${\overset{⃑}{F}}_{att}(ovs)$ along the X and Y axes are defined by Equations (11) and (12):

(11) $$F_{att\_x}(ovs)=F_{att}(ovs)\cos(\theta_{att}(ao)),$$

(12) $$F_{att\_y}(ovs)=F_{att}(ovs)\sin(\theta_{att}(ao)),$$

where *θ_att_*(*ao*) represents the angle between the AMR and the line connecting it to the center of the obstacle. After the AMR passes the virtual subgoal, since the obstacle no longer poses a collision threat, the area occupied by this obstacle is removed, and the attraction force generated by the goal point guides the AMR’s movement.

#### 2.2.3. Angle Constraints

To minimize the turning angle and reduce the angular jitter and abrupt changes in the AMR’s direction, aiming for a smoother path, the concept of angle constraint was introduced in this study [30,36,37].

The maximum desired turning angle *θ_ide_* was defined based on the structural characteristics of the AMR itself, and the actual heading angle change ∆*θ_com_* was defined by Equation (13):

(13) $$\Delta \theta_{\mathrm{co}m}=\theta_{t+1}-\theta_{t},$$

where *θ_t+_*_1_ represents the turning angle of the AMR at the next moment and *θ_t_* represents the current angle of the AMR. To decrease the turning angle of the AMR, the turning angle is limited. The actual turning angle of the AMR at the next moment *θ_act_* is defined as shown in Equation (14):

(14) $$\theta_{act}=\left\{\begin{array}{l} \theta_{t+1}\begin{array}{c} \begin{array}{c} \begin{array}{c} \begin{array}{c} \;\;\;\;\;\;\;\;\;\;\;\left|\Delta \theta_{com}\right|\leq \theta_{ide} \end{array} \end{array} \end{array} \end{array}\\ \mathrm{sgn}(\Delta \theta_{com})\Delta \theta_{ide}\begin{array}{cc} & \left|\Delta \theta_{com}\right|>\theta_{ide} \end{array} \end{array}\right.,$$

## 3. Algorithm Simulation and Result Analysis

Considering the impact of energy consumption power *P_AMR_* by the AMR during the entire period T, this paper introduced energy consumption to evaluate the simulation results. The formula for calculating *E_AMR_* energy consumption is shown in Equation (15) [40]:

(15) $$E_{AMR}=\sum_{i}P_{AMR}(i)t_{i}\begin{array}{c} \;(i=1,2,3\cdots n) \end{array}$$

where *t_i_* represents the i moment during the entire period T.

### 3.1. OVS Selection

**Figure 8:**
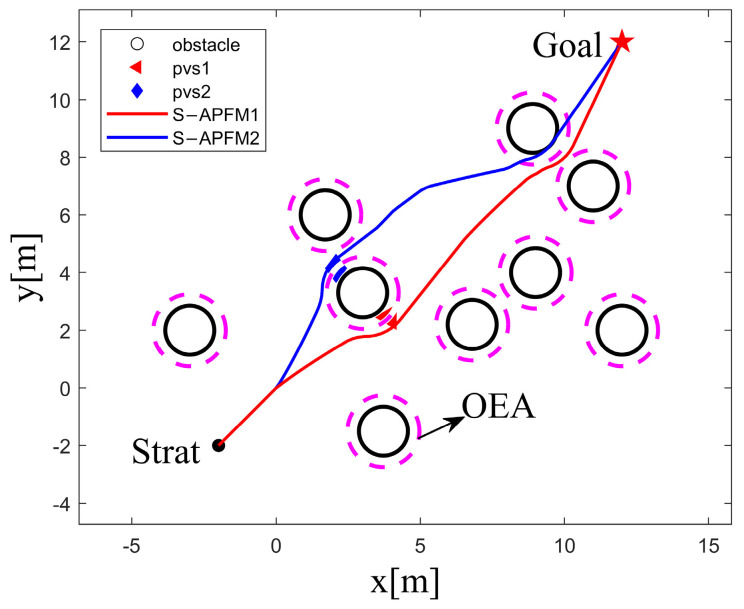
Influence diagram of different PVS.

In the case of selecting OVS in the PVS, the coordinates of the starting point and goal point of the AMR were set as (−2, −2) and (12, 12), respectively. The coordinates of the main obstacles were (3, 3.3), as shown in Figure 8.

When the AMR selects PVS1 as an OVS, it only needs to avoid obstacle (3, 3.3) during the driving process. When the AMR selects PVS2 as an OVS, it not only needs to avoid obstacle (3, 3.3) but also needs to avoid obstacle (8.3,9.9) during the driving process. Additionally, obstacle avoidance actions are added throughout the entire path, which increases energy consumption.

The experimental data for selecting PVS1 or PVS2 as OVS is shown in Table 1. Choosing PVS1, with the smaller J value, results in reductions in algorithm iterations, energy consumption, and path length compared to choosing PVS2. Therefore, selecting OVS with a smaller J value can reduce the impact of obstacles on the subsequent path of the AMR.

### 3.2. Simulation Tests in Different Environments

In this section, simulation experiments were mainly conducted on three scenarios: local minima, unreachable goals, and complex environments. The traditional artificial potential field method (referred to as TAPF), the algorithm in reference [22], the algorithm in reference [28] (referred to as IM-APF), and the proposed algorithm in this paper (referred to as S-APFM) were simulated and compared.

#### 3.2.1. Local Minimum

**Figure 9:**
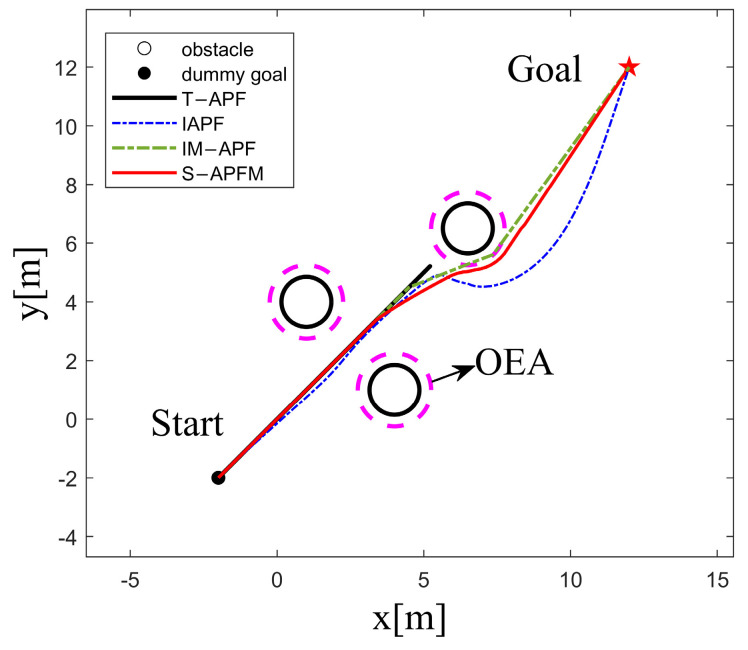
Local minimum simulation path diagram.

For the scenario of local minima, the starting point of the AMR was set at (2, 2) and the goal point coordinates were (12, 12). The coordinates of the obstacle were (6.5, 6.5), as shown in Figure 9. When the AMR moved towards the obstacle using the T-APF algorithm, it got stuck in front of the obstacle. In the other algorithms, due to the presence of virtual subgoals, the AMR was able to escape from the local minima and reach the goal point when it became trapped in a local minimum situation.

**Figure 10:**
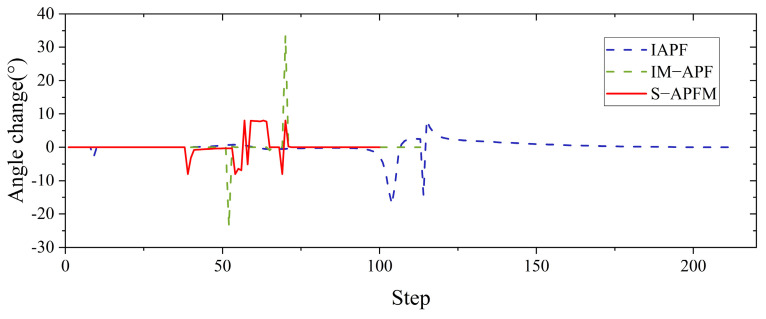
Local minimum simulation angle change diagram.

Comparing the angle changes of different algorithms, as shown in Figure 10, the heading angle variances for IM-APF algorithms was 14.87, respectively, while the heading angle variance for the S-APFM algorithm was 8.15.

Further statistics on the number of iterations, energy consumption, and path lengths for different algorithms in the scenario of local minima are presented in Table 2. In terms of the number of iterations, the S-APFM algorithm showed an improvement of 52.58% and 11.40% in efficiency compared to the IAPF algorithm and IM-APF algorithm, respectively. This indicates that the S-APFM algorithm has higher computational efficiency. In terms of energy consumption, the S-APFM algorithm reduced energy consumption by 52.56% and 11.39% compared to the IAPF algorithm and IM-APF algorithm, respectively. This demonstrates that the S-APFM algorithm has lower energy consumption during the AMR’s movement. As for path length, the simulated path length of the S-APFM algorithm was only slightly different from that of the IM-APF algorithm, but it decreased by 6.16% compared to the IAPF algorithm.

#### 3.2.2. Unreachable Goal

**Figure 11:**
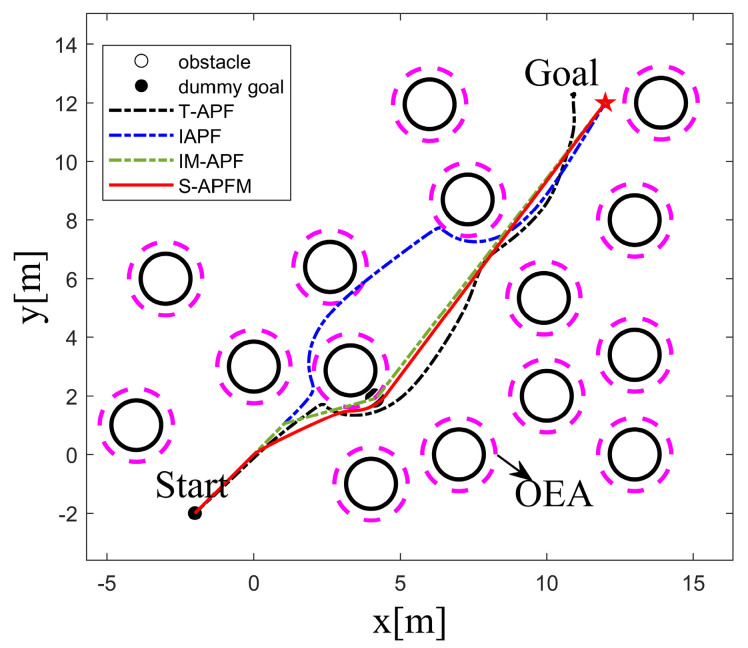
Unreachable goal simulation path diagram.

In the scenario of an unreachable goal, the starting point of the AMR was set at (−2, −2), and the goal point was located at (12, 12), as shown in Figure 11. When the AMR moved towards the goal point using the T-APF algorithm, it got stuck in front of the obstacle (13.9, 12) because the repulsive force from the obstacle was greater than the attractive force from the goal point, preventing it from reaching the goal. On the other hand, the IAPF algorithm, IM-APF algorithm, and S-APFM algorithm were able to ignore irrelevant obstacles and reach the goal point.

**Figure 12:**
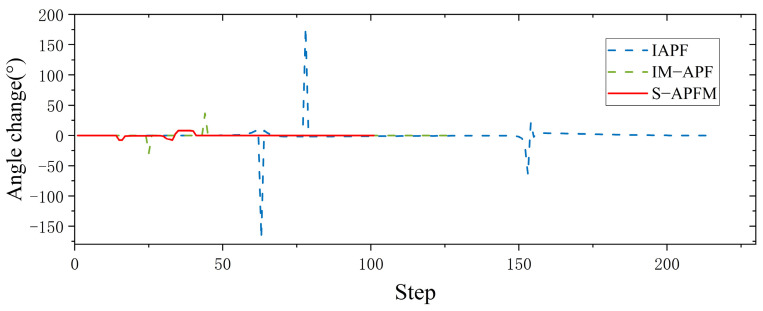
Unreachable goal simulation angle change diagram.

Comparing the angle changes for different algorithms, as shown in Figure 12, both the IAPF algorithm and the IM-APF algorithm adjusted their heading angles by more than 20 degrees during obstacle avoidance. In contrast, the S-APFM algorithm kept the heading angle deviation within 10 degrees while the AMR was moving, indicating that the S-APFM algorithm had more optimized path planning.

Further statistics on the number of iterations, energy consumption, and path length for different algorithms in the scenario of an unreachable goal are presented in Table 3. The S-APFM algorithm reduced energy consumption by 52.97% and 12.05% and the number of steps by 52.97% and 12.05%, respectively, compared to the IAPF al-gorithm and IM-APF algorithm during obstacle avoidance. Additionally, the planned path length of the S-APFM algorithm was slightly reduced compared to the IAPF al-gorithm and IM-APF algorithm.

#### 3.2.3. Complex Environments

**Figure 13:**
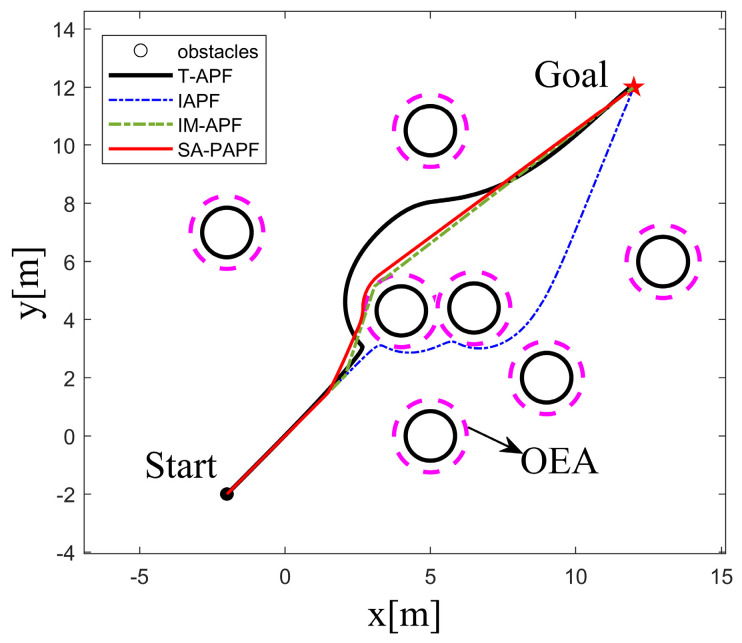
Complex environment simulation path diagram.

To validate the feasibility of the S-APFM algorithm in complex environments, this study created a simulated environment for testing and verification. The starting point of the AMR was set at (−2, −2), and the endpoint was (12, 12). The obstacles that affected the path were located at (4, 4.3) and (6.5, 4.4), as shown in Figure 13. In the face of complex scenarios, the local path planning of the S-APFM algorithm was able to successfully avoid obstacles and select an appropriate path to reach the goal point. Similarly, the global path planning of the IAPF algorithm, IM-APF algorithm, and T-APF algorithm were also able to reach the goal point.

**Figure 14:**
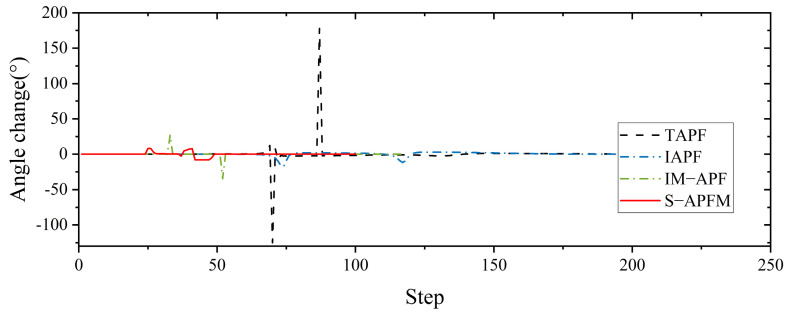
Complex environment simulation angle change diagram.

Comparing the angle variations corresponding to different algorithms, as shown in Figure 14, it can be observed that the T-APF algorithm and IAPF algorithm exhibited significant angle changes when encountering obstacles. The T-APF algorithm had a maximum turning angle of 177 degrees, while the IAPF algorithm had a maximum turning angle of 17.53 degrees. Additionally, although the IM-APF algorithm had fewer changes in heading angle along the travel path, it reached a maximum turning angle of 34.37 degrees, resulting in an uneven path. In contrast, the S-APFM algorithm, with its zone-based predictive potential field model, restricted the angle changes to small an-gles (within 10 degrees), resulting in smoother travel paths. The variance in the head-ing angle changes for the four algorithms was 211.66, 6.58, 16.38, and 7.12, respectively. From the variance data, it can be inferred that the S-APFM algorithm had a smaller variance in heading angle changes, indicating less fluctuation in angle variations.

Further statistics on the iteration times, energy consumption, and path length of different algorithms in complex environments are presented in Table 4. The S-APFM algorithm, utilizing the optimal virtual subgoal model and zone-based predictive potential field model, selects a reasonable path and smoothens the trajectory. Compared to the IAPF algorithm and IM-APF algorithm, the S-APFM algorithm reduced iteration times by 53.88% and 14.41% and decreased energy consumption by 53.87% and 14.39%. Simultaneously, it planned the shortest paths in complex scenarios. This indicates that the S-APFM algorithm was able to effectively reduce the energy consumption and path length of the robot during obstacle avoidance, thereby improving the robot’s movement efficiency and autonomy.

In local path planning, when facing complex situations, the S-APFM algorithm used detection distance to gather information about obstacles ahead and selected relatively reasonable paths based on predicted potential field forces and angle adjustment strategies, thereby reducing the number of turns. Additionally, due to the presence of the virtual subgoal utility function, the robot was able to intelligently avoid obstacles and select shorter paths. In global path planning, the IAPF algorithm and TAPF algorithm experienced path curvature and increased path length and energy consumption when faced with two obstacles at similar distances. Although the IM-APF algorithm was able to obtain reasonable paths from global information, it tended to result in path deviations near the virtual subgoal.

## 4. Experimental Results and Analysis

**Figure 15:**
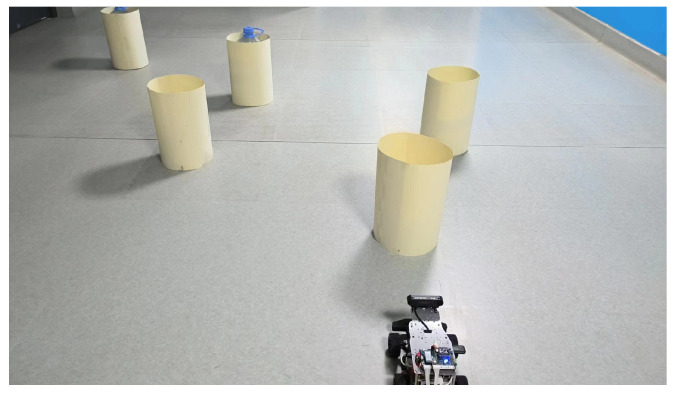
The AMR and physical environment maps.

**Figure 16:**
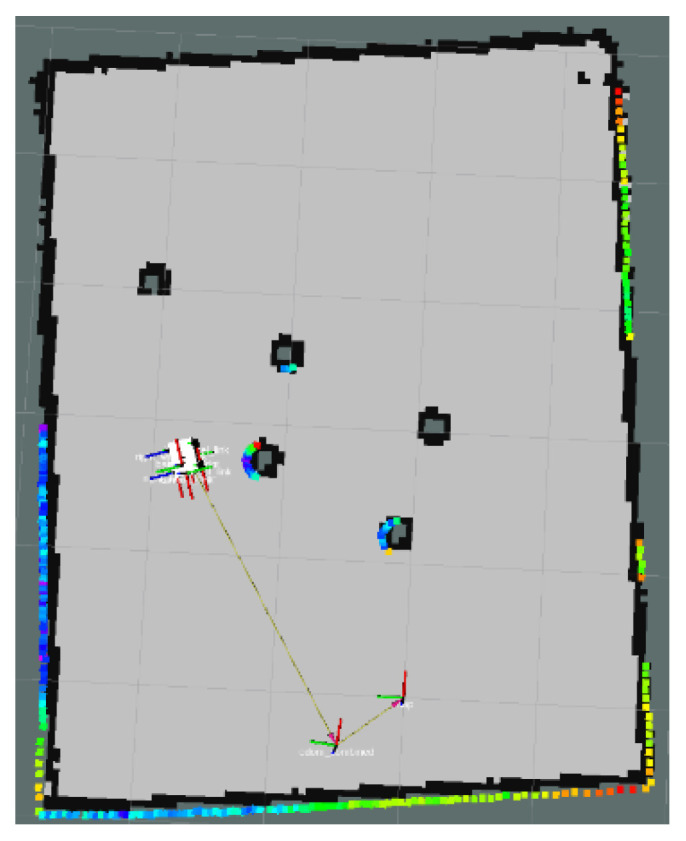
Physics experiment environmental scan map.

This article created an AMR task execution environment with multiple obstacles and conducted physical experiments for three scenarios in Section 4. An environment map was established for the AMR’s adaptive Monte Carlo localization. The AMR and physical environment maps are shown in Figure 15 and the physical environment creation map is shown in Figure 16.

### 4.1. Local Minimum Test

**Figure 17:**
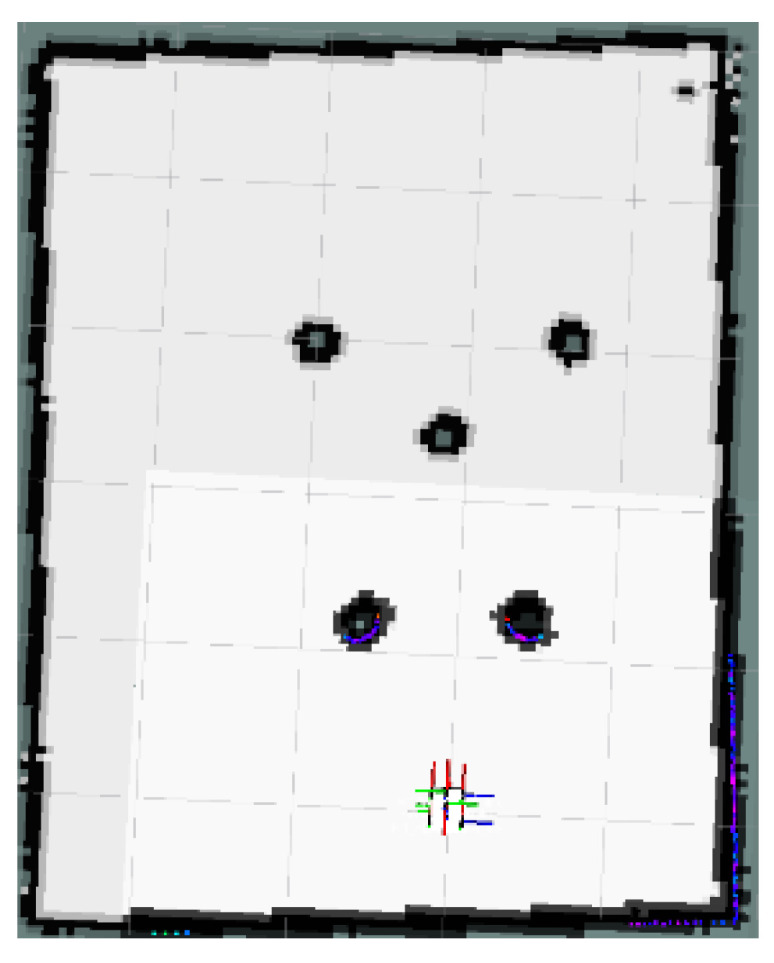
Local minimum scenario map.

**Figure 18:**
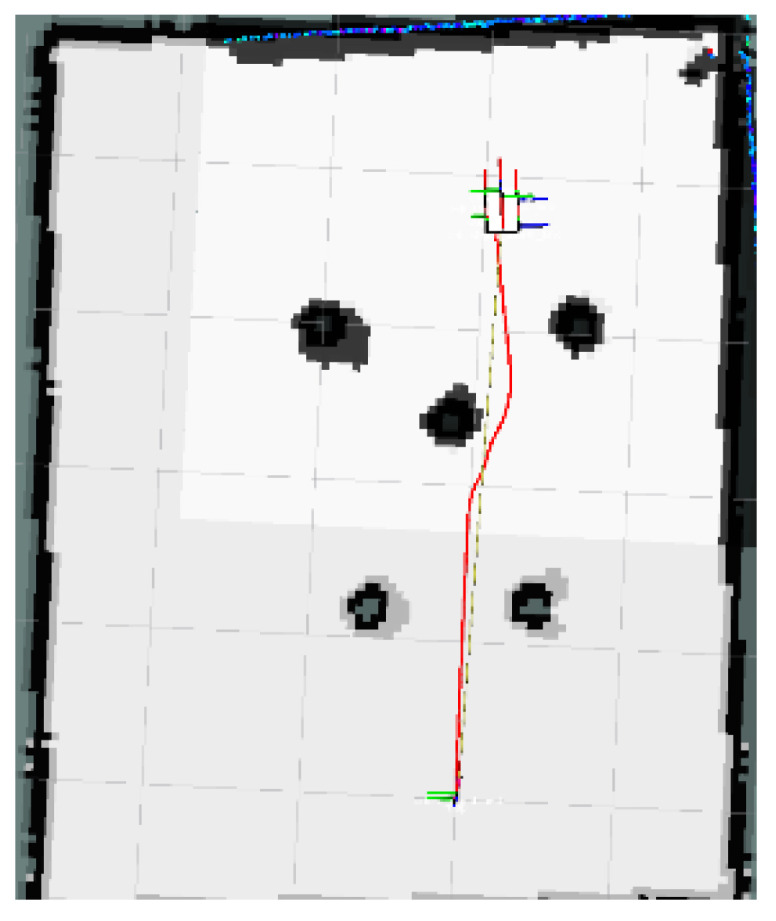
S-APFM path in local minimum scenario.

**Figure 19:**
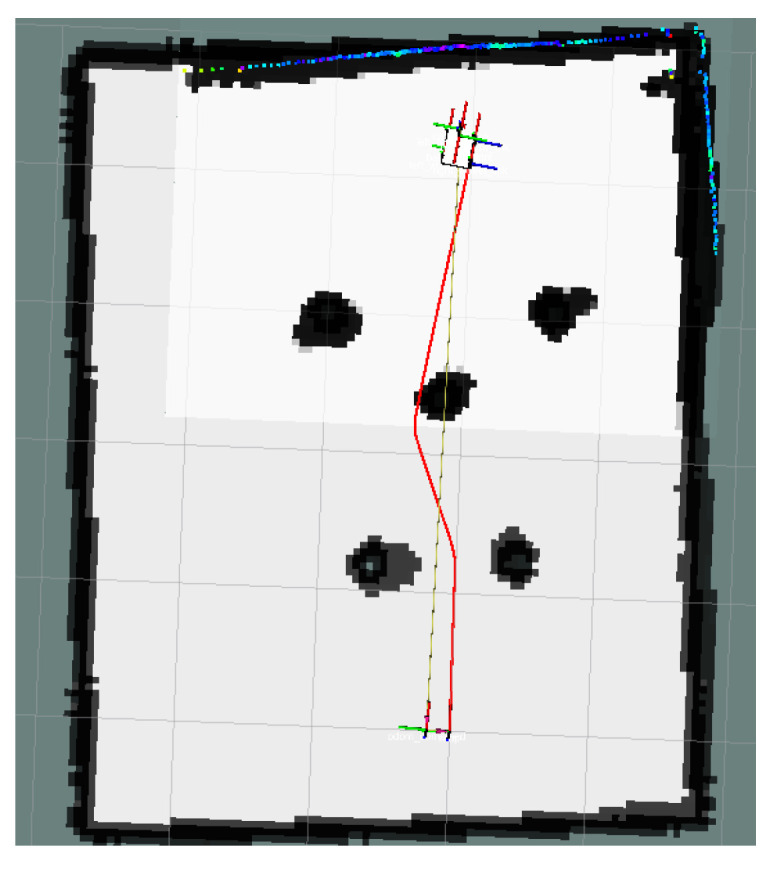
IM-APF path in local minimum scenario.

**Figure 20:**
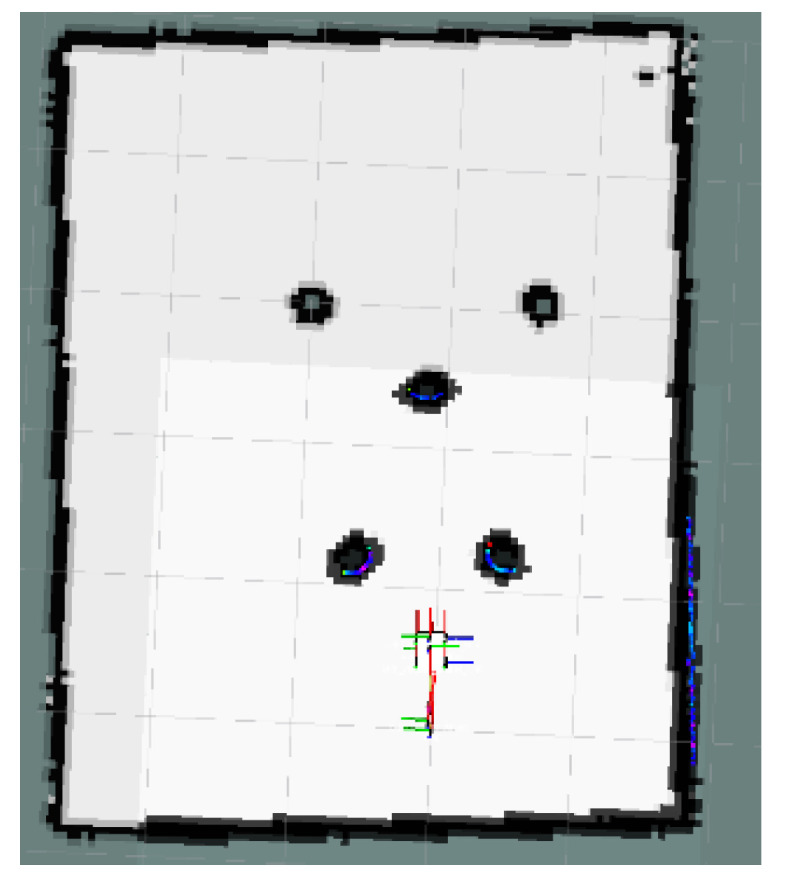
TAPF path in local minimum scenario.

The global map for the local minimum scenario is shown in Figure 17. The local path planning path for the AMR under the S-APFM algorithm is depicted in Figure 18, while the global path planning paths under the IM-APF algorithm and TAPF algorithm are shown in Figure 19 and Figure 20, respectively. When facing different types of local minimum scenarios, both the S-APFM algorithm and the IM-APF algorithm were able to escape the local minimum traps and successfully reach the destination point. However, the TAPF algorithm got stuck in front of the obstacle due to its inability to overcome the influence of local minima.

**Figure 21:**
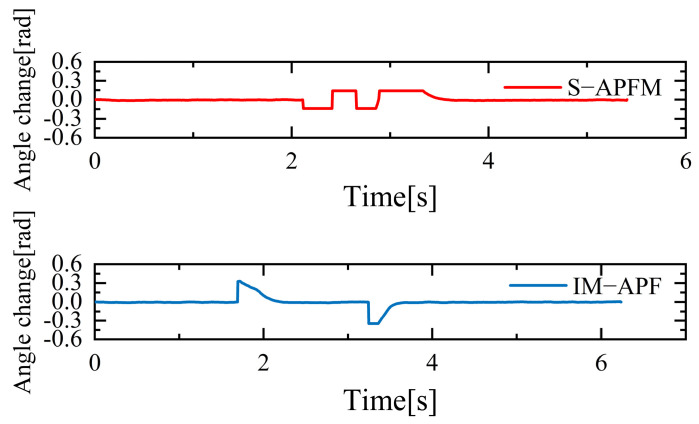
Local minimum angle radian change diagram.

**Figure 22:**
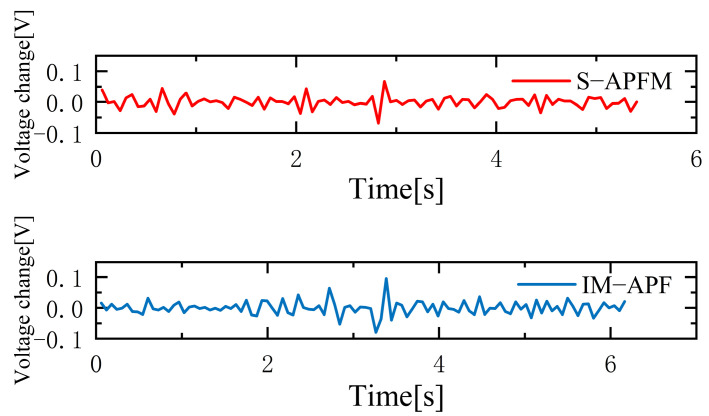
Under simulation local minimum voltage variation diagram.

The change in the cornering angle in radians during AMR travel was counted and is shown in Figure 21. The variance in the turning angle changes for the S-APFM algorithm and the IM-APF algorithm was 0.0052 and 0.006, respectively. This indicates that the S-APFM algorithm had smaller turning angle variations and smoother angle changes. The voltage variation of the battery during this movement was also monitored and is depicted in Figure 22. The sum of the squared voltage variations for the two algorithms was 0.0385 and 0.040, respectively, indicating that the S-APFM algorithm consumed less battery energy.

The data for the time and length spent by the AMR in the physical experiments are presented in Table 5. In terms of AMR travel time, the S-APFM algorithm showed a 11.87% improvement compared to the IM-APF algorithm, while the path length increased by 0.47% during actual operation. 

When faced with local minima situations, the S-APFM algorithm was able to smoothly escape from local minima points and reach the goal point more quickly.

### 4.2. Unreachable Goal Test

**Figure 23:**
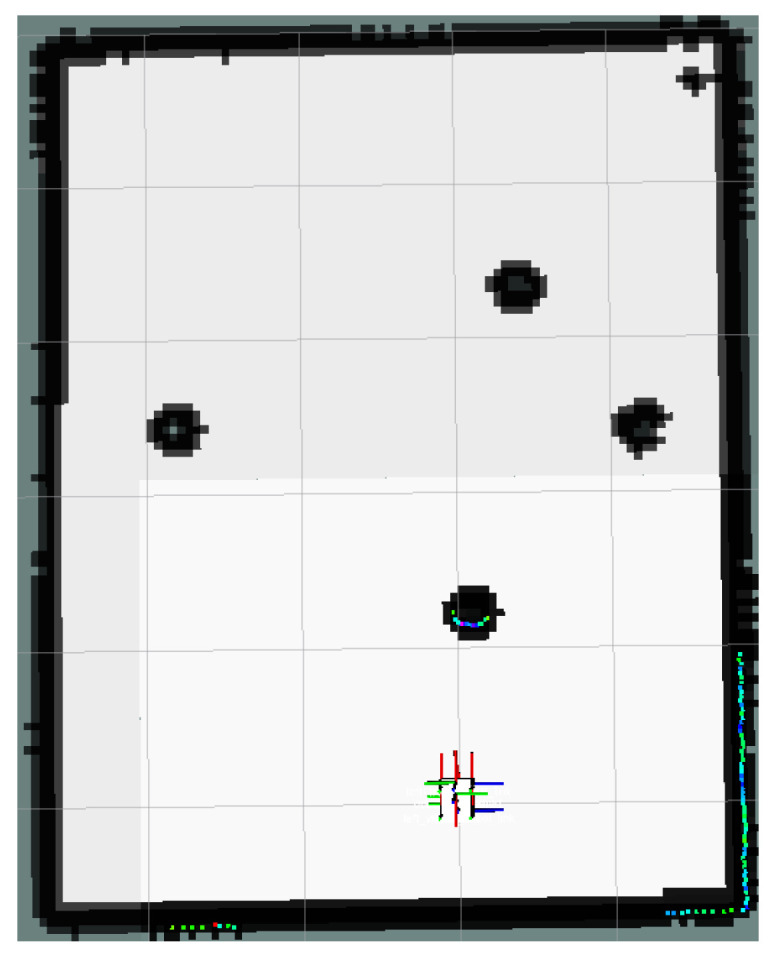
Unreachable goal scenario map.

**Figure 24:**
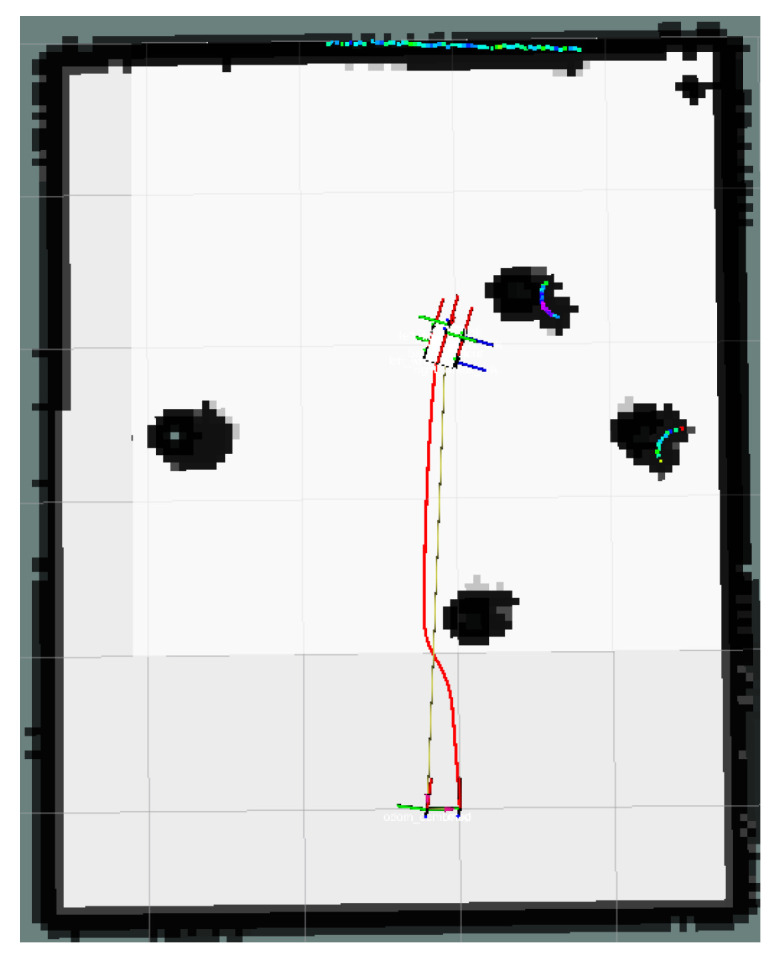
S-APFM path at unreachable goal scenario.

**Figure 25:**
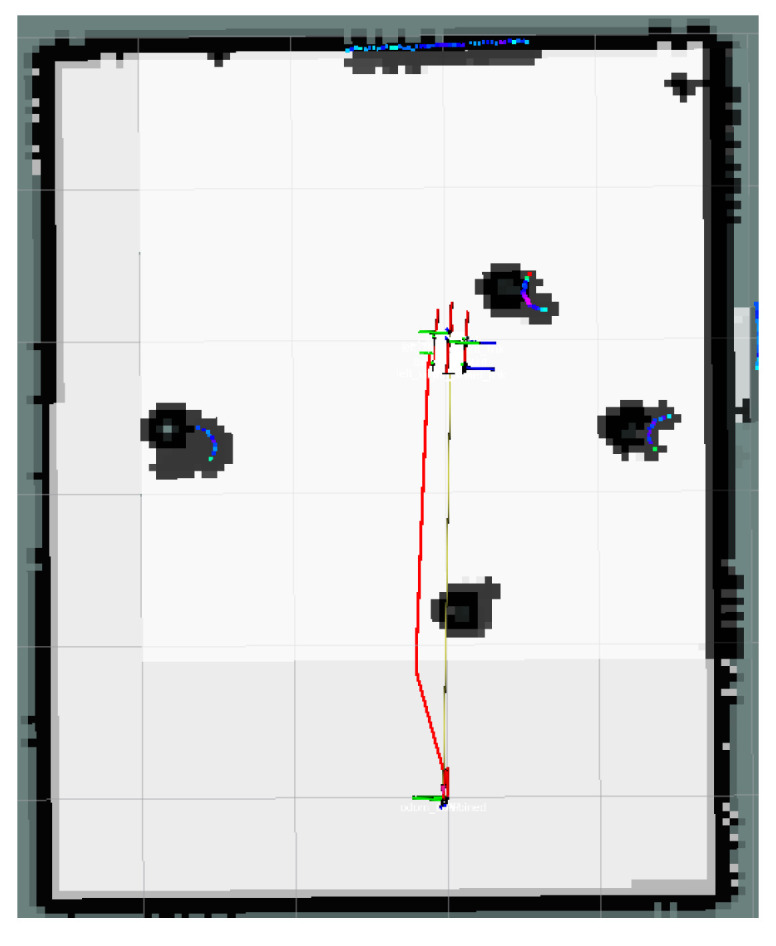
IM-APF path at unreachable goal scenario.

**Figure 26:**
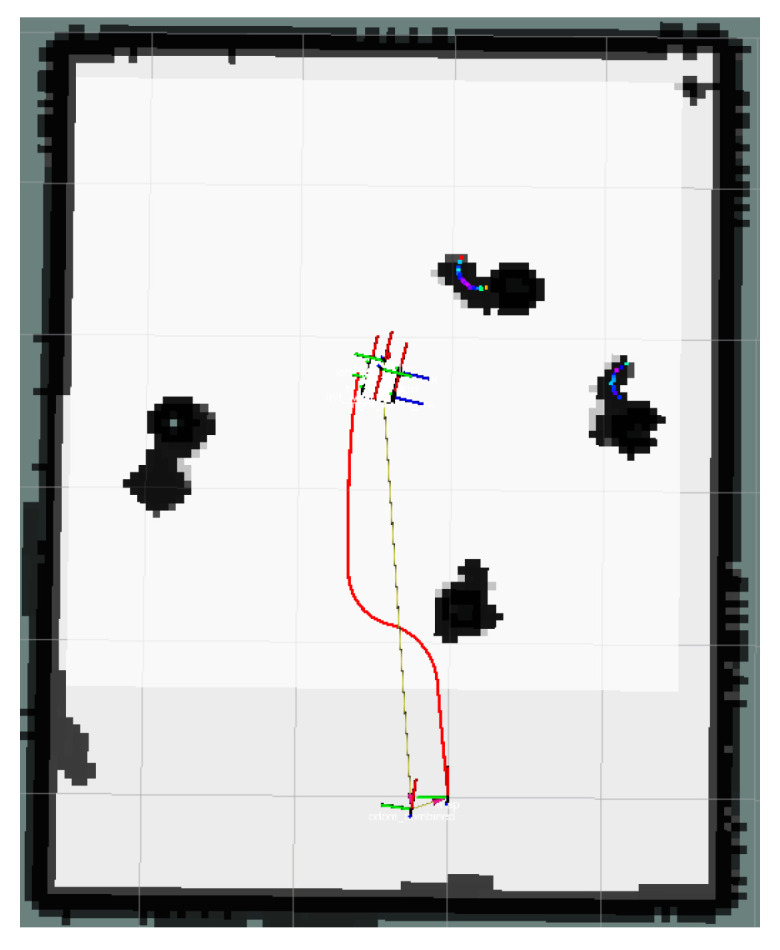
TAPF path at unreachable goal scenario.

In Figure 23, the global map of the goal unreachable scenario is presented, where the goal point is located inside the OIA of an obstacle. The local path planning path under the S-APFM algorithm is shown in Figure 24, and the global path planning paths under the IM-APF and TAPF algorithms are shown in Figure 25 and Figure 26. The S-APFM algorithm and IM-APF algorithm can avoid the goal being unreachable due to the goal point being inside the OIA of obstacles, while the TAPF algorithm tends to linger around the goal point because it experiences a repulsive force greater than the attractive force near the goal point.

**Figure 27:**
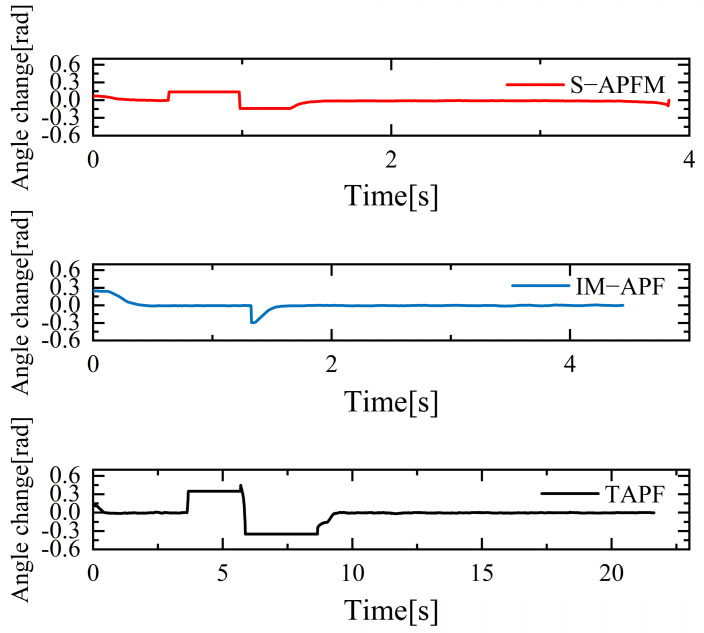
Unreachable goal angular radian change diagram.

**Figure 28:**
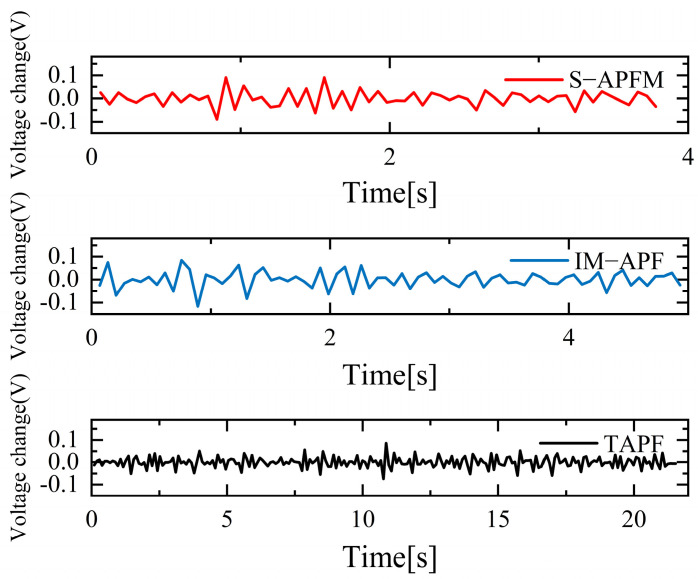
Diagram of voltage variation in Unreachable goal situation.

The corner changes in radians during path travel were counted, as shown in Figure 27. The variance in the angular changes for the S-APFM algorithm and the IM-APF algorithm was 0.005 and 0.0045, respectively. Although the IM-APF algorithm had a smaller variance in angular changes during obstacle avoidance compared to the S-APFM algorithm, its planned path exhibited a sudden change in angle, rendering the path non-smooth. Statistical battery voltage changes during driving are shown in Figure 28. The sum of the squared battery voltage changes for the S-APFM algorithm, IM-APF algorithm, and TAPF algorithm was 0.088, 0.095, and 0.118, respectively, indicating that the S-APFM algorithm consumed less battery energy during the traversal process.

The data for path traversal time and path length in physical experiments with AMR are presented in Table 6. In terms of AMR travel time, the S-APFM algorithm exhibited a 11.77% improvement compared to the IM-APF algorithm, while the path length increased by 0.33% during actual travel.

When faced with the scenario of an unreachable goal, the S-APFM algorithm remained practical and effective. 

### 4.3. Complex Environments Test

**Figure 29:**
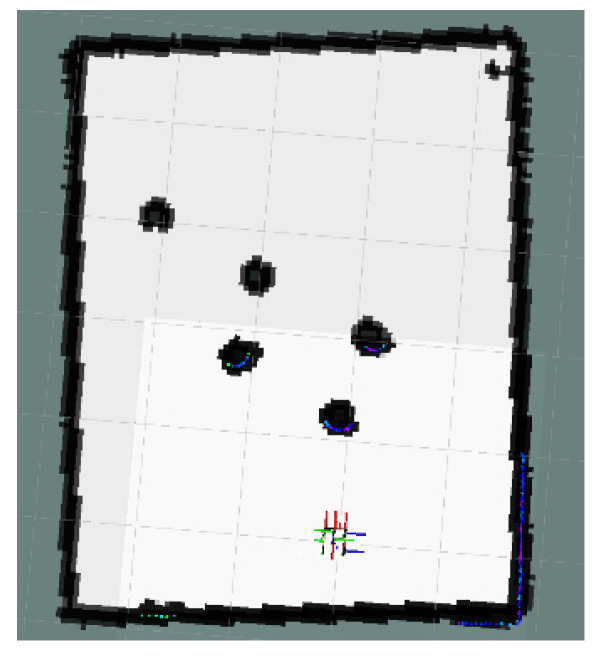
Complex environments scenario map.

**Figure 30:**
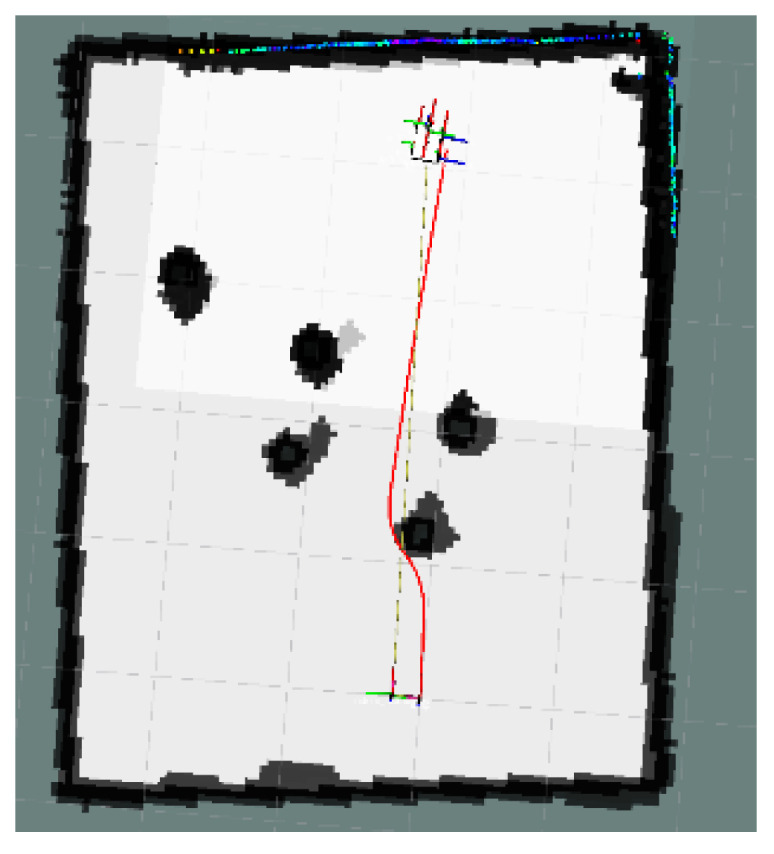
S-APFM path in Complex environments scenario.

**Figure 31:**
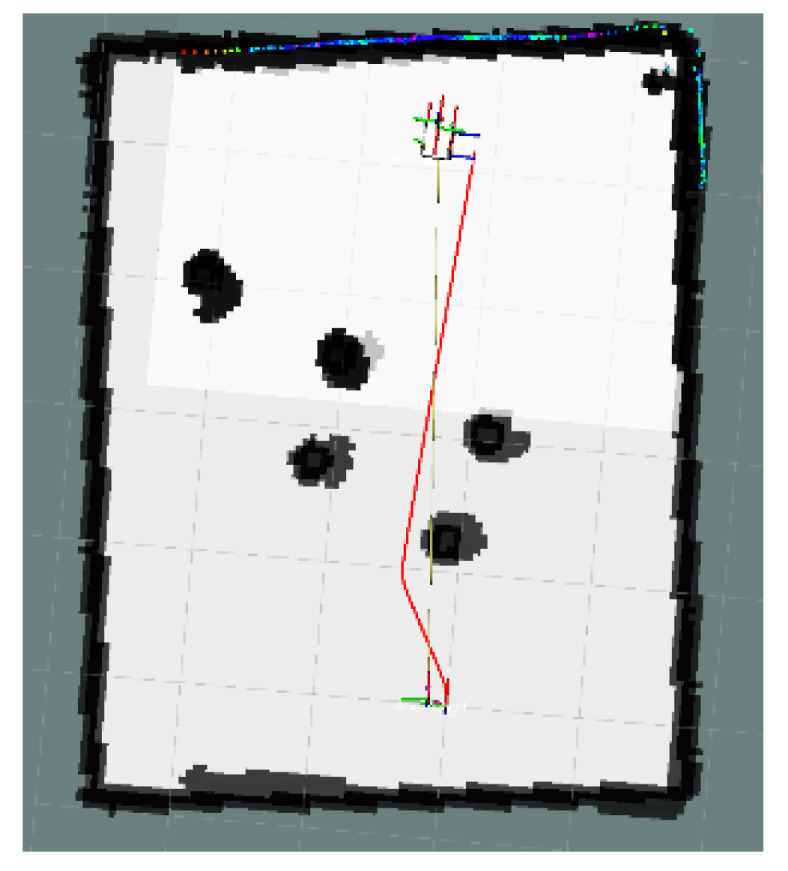
IM-APF path in Complex environments scenario.

**Figure 32:**
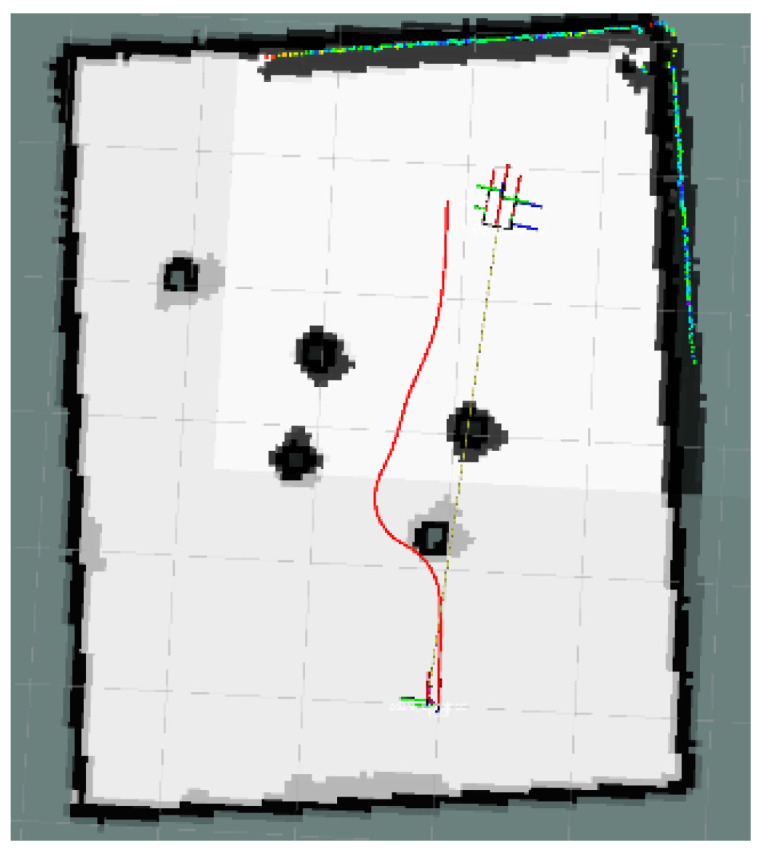
TAPF path in Complex environments scenario.

This section focuses on the physical experiments conducted for the simulated simulation in Section 3.2.3 of this paper. The experimental map is shown in Figure 29. The local path planning path under the S-APFM algorithm is illustrated in Figure 30, while the global path planning paths under the IM-APF algorithm and TAPF algorithm are shown in Figure 31 and Figure 32. When employing the S-APFM algorithm for local path planning in complex environments, it was still able to select an optimal velocity profile to reach the goal point, resulting in a smoother path compared to the paths produced by the IM-APF and TAPF algorithms for obstacle avoidance. Under global path planning, the IM-APF algorithm was capable of reaching the goal point, but the TAPF algorithm generated excessively large turning angles due to the repulsive forces caused by obstacles and also failed to reach the goal point, as the gravitational force gradually diminished as the AMR approached the goal.

**Figure 33:**
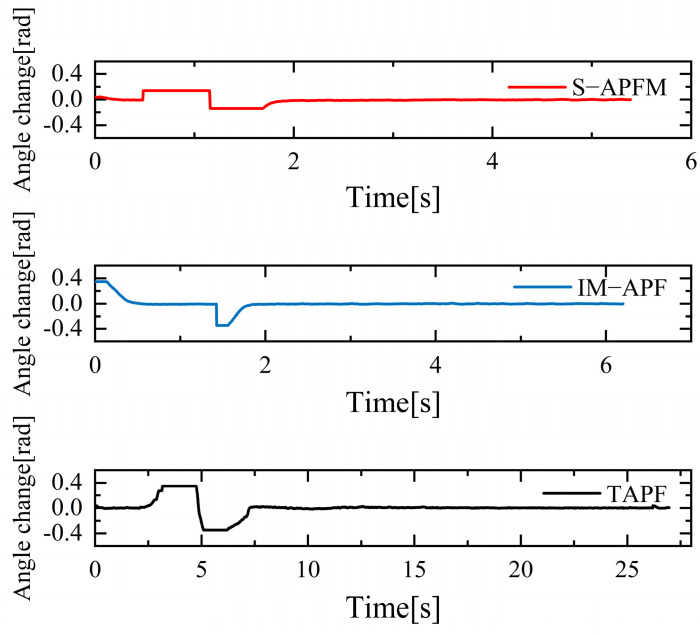
Diagram of radian changes in complex environmental situations.

The corner change in radians during the statistical path traveling is shown in Figure 33. The S-APFM algorithm was able to limit the angular velocity within a certain range during traveling, while the IM-APF algorithm and the TAPF algorithm were constrained by the angular limitation of the robot’s structure, and were not able to avoid obstacles by the corner radians in the simulation test. For the S-APFM algorithm, IM-APF algorithm, and the TAPF algorithm, the variance of the corner change of the three algorithms was 0.0056, 0.008, and 0.016, respectively, suggesting that the S-APFM algorithm may be better adapted to generate smoother paths when the robot’s structure has angular constraints.

**Figure 34:**
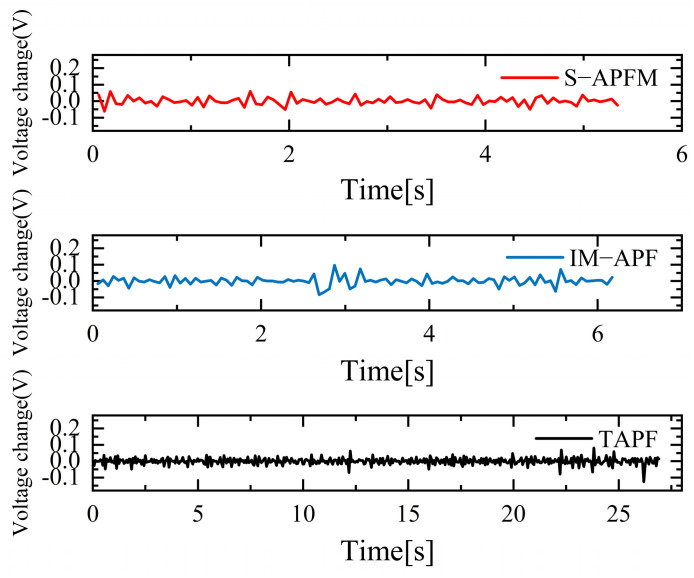
Diagram of voltage change in a complex environment situations.

The statistics on battery voltage changes during traveling are shown in Figure 34. When the S-APFM algorithm was driving, due to the reduction in the angle of mutation, the AMR battery voltage was changed compared to the IM-APF algorithm, and compared to the TAPF algorithm, the transformation amplitude was decreased. For the S-APFM algorithm, IM-APF algorithm, and TAPF algorithm, the square sum of the battery voltage change was 0.054, 0.061, 0.179, respectively, indicating that the S-APFM algorithm maintained low power energy consumption when facing complex environments.

The results of AMR in physical experiments on path consumption time and path length data are shown in Table 7. AMR can be based on the value of the benefit function, derived from the different virtual subgoals in the local area of the path value; a smaller value of the benefit function indicates that the AMR are moving toward the current virtual subgoals on a forward path with less obstacle avoidance action, with less energy consumed. Although the path length in the IM-APF algorithm was 0.71% less than that in the S-APFM algorithm, it improved 9.23% compared to the IM-APF algorithm in terms of traveling time. In the complex environment, the S-APFM algorithm performed better in local path planning.

## 5. Discussion

The traditional artificial potential field and its improved versions have certain limitations when dealing with issues such as local minima, unreasonable paths, and sudden changes in heading angle. In the face of local minima and unreachable goal situations, the traditional artificial potential field gradually reduces the value of attraction as the AMR approaches the goal point, causing the AMR to fail to reach the goal point. The improved artificial potential field utilizes virtual subgoals to reach the goal point, but when turning towards a virtual subgoal, the AMR tends to have a larger turning angle. Additionally, due to the turning radius and angle limitations of the Ackermann-type AMR structure, it cannot suddenly turn at a large angle during travel, which leads to a certain collision probability and an uneven path. This study aims to propose an efficient local path planning algorithm for the obstacle avoidance system of AMR during task execution. Simulation experiments and physical experiments on AMR show that the proposed algorithm can achieve fast, safe, and reasonable path planning in different scenarios. Compared with traditional and improved algorithms, the proposed algorithm significantly improves the driving time, path turning angle, and path rationality. The experimental results show that the proposed algorithm can generate reasonable paths even in complex environments and local minima. Compared with the improved artificial potential field, the proposed algorithm improved the driving time by 9.23%. Moreover, the variance of the heading angle change during travel was smaller and the path was smoother, enabling the AMR to reach the destination faster and safer. The path planning algorithm proposed in this study has significant practical significance for AMR automatic driving systems; it can help AMR plan the best path quickly and safely, improve driving efficiency, and reduce collision risks. Additionally, the proposed algorithm can also be applied in fields such as drone control and logistics management, providing support for the development of intelligent transportation and logistics systems. Although the local path planning algorithm performs well in multiple test scenarios, there are still some limitations; for example, the algorithm may be limited in extremely complex obstacle environments. Future research can explore more advanced obstacle avoidance models to address these challenges. In addition, our algorithm can be further improved to support the avoidance of dynamic obstacles.

## 6. Conclusions

This paper presented a local path planning method for autonomous AMR under the predictive potential field to address the issues of traditional artificial potential field algorithms. This method introduced virtual subgoals to guide the robot to avoid stagnant waypoints and overcome local minimum problems. To smooth the heading angle change during obstacle avoidance, a predictive potential field with virtual subgoals was introduced. Additionally, to address the issue of unreasonable robot paths in local path planning, a utility function for virtual subgoals was proposed, which considered both obstacle distance and angle factors. Finally, to ensure that the robot can travel at a faster speed, constraints on turning angles were added. The feasibility of the algorithm was demonstrated through simulation experiments and experiments on the ROS platform. This study improved upon the shortcomings of traditional artificial potential field algorithms and proposed several key technical innovations: Firstly, the concept of virtual subgoals was introduced to guide the robot’s actions, solving the problems of unreachable goals and local minimums. Secondly, by combining the predictive potential field and virtual subgoals, smooth turns during obstacle avoidance were achieved, improving the effectiveness of path planning. Furthermore, by introducing a utility function for virtual subgoals and considering factors such as obstacle distance and angle, the issue of unreasonable robot paths in local path planning was effectively addressed. Lastly, constraints on turning angles were added to ensure fast robot travel while maintaining safety. The results of simulation experiments and experiments on the ROS platform demonstrated the effectiveness and feasibility of this method in solving the autonomous AMR path planning problem. Future research directions will be to further optimize the algorithm, improve the efficiency and accuracy of path planning, and apply the algorithm to a wider range of autonomous AMR fields.

## Figures and Tables

**Table 1:** Comparison of simulation data of different PVS.

Pending Virtual Subgoals	*J*	Iteration Number (N)	Energy Consumption (KJ)	Path Length (m)
PVS1	7.30	102	10.47	19.80
PVS2	76.60	107	11.10	21.00

**Table 2:** Comparison data of Local minimum simulation.

Scenarios	Algorithm	Iteration Number (N)	Energy Consumption (KJ)	Path Length (m)
Local minimum	T-APF	-	-	-
	IAPF	213	22.07	21.10
	IM-APF	114	11.82	19.93
	S-APFM	101	10.47	19.80

**Table 3:** Comparison data of unreachable goal algorithms.

Scenarios	Algorithm	Iteration Number (N)	Energy Consumption (KJ)	Path Length (m)
Unreachable goal	T-APF	-	-	-
	IAPF	217	22.48	21.24
	IM-APF	116	12.02	20.06
	S-APFM	102	10.58	20.00

**Table 4:** Comparison data of complex environment algorithms.

Scenarios	Algorithm	Iteration Number (N)	Energy Consumption (KJ)	Path Length (m)
Complex environment	T-APF	216	22.38	21.40
	IAPF	219	22.69	21.41
	IM-APF	118	12.23	19.99
	S-APFM	101	10.47	19.80

**Table 5:** Comparison of experimental data under local minimum.

Scenarios	Algorithm	Time (s)	Path Length (m)	Situation
Local minima	T-APF	-	-	Failure
	IM-APF	6.23	4.24	Success
	S-APFM	5.49	4.22	Success

**Table 6:** Comparison of experimental data under unreachable goal.

Scenarios	Algorithm	Time (s)	Path Length (m)	Situation
Unreachable goal	T-APF	21.634	3.460	Failure
	IM-APF	4.443	3.034	Success
	S-APFM	3.920	3.024	Success

**Table 7:** Comparison of experimental data under Complex environment situations.

Scenarios	Algorithm	Time (s)	Path Length (m)	Situation
Complex environment	T-APF	-	-	Failure
	IM-APF	6.07	4.20	Success
	S-APFM	5.51	4.23	Success

## Data Availability

The data used in the experimental evaluation of this study are available within this article.
